# The structure of the quality of clinical practice guidelines with the items and overall assessment in AGREE II: a regression analysis

**DOI:** 10.1186/s12913-019-4532-0

**Published:** 2019-11-04

**Authors:** Yosuke Hatakeyama, Kanako Seto, Rebeka Amin, Takefumi Kitazawa, Shigeru Fujita, Kunichika Matsumoto, Tomonori Hasegawa

**Affiliations:** 1 Department of Social Medicine, Toho University School of Medicine, 5-21-16, Omori-Nishi, Ota-ku, Tokyo, 143-8540 Japan; 2 Department of Social Medicine, Toho University Graduate School of Medicine, Tokyo, Japan; 3 Faculty of Health Sciences, Tokyo Kasei University, Saitama, Japan

**Keywords:** Practice guideline, Practice guidelines as topic, AGREE, Quality, Appraisal

## Abstract

**Background:**

The Appraisal of Guidelines for Research & Evaluation (AGREE) II has been widely used to evaluate the quality of clinical practice guidelines (CPGs). While the relationship between the overall assessment of CPGs and scores of six domains were reported in previous studies, the relationship between items constituting these domains and the overall assessment has not been analyzed. This study aims to investigate the relationship between the score of each item and the overall assessment and identify items that could influence the overall assessment.

**Methods:**

All Japanese CPGs developed using the evidence-based medicine method and published from 2011 to 2015 were used. They were independently evaluated by three appraisers using AGREE II. The evaluation results were analyzed using regression analysis to evaluate the influence of 6 domains and 23 items on the overall assessment.

**Results:**

A total of 206 CPGs were obtained. All domains and all items except one were significantly correlated to the overall assessment. Regression analysis revealed that Domain 3 (Rigour of Development), Domain 4 (Clarity of Presentation), Domain 5 (Applicability), and Domain 6 (Editorial Independence) had influence on the overall assessment. Additionally, four items of AGREE II, clear selection of evidence (Item 8), specific/unambiguous recommendations (Item 15), advice/tools for implementing recommendations (Item 19), and conflicts of interest (Item 22), significantly influenced the overall assessment and explained 72.1% of the variance.

**Conclusions:**

These four items may highlight the areas for improvement in developing CPGs.

## Introduction

Clinical practice guidelines (CPGs) are statements that include recommendations based on “a systematic review of evidence and an assessment of the benefits and harms of alternative care options” for assisting “practitioner and patient decisions” [1, 2]. Additionally, CPGs have been shown to improve clinical outcomes [3–16].

Numerous development manuals and over 40 appraisal tools have been published to ensure the quality of CPGs [17, 18]. The most widely applied and validated CPG assessment tool is the Appraisal of Guidelines for Research and Evaluation (AGREE) II [19]. AGREE II was published in 2009 as a revised version of the original AGREE issued in 2001 [20] and is composed of 23 items grouped into 6 domains and 2 overall CPG assessment items (Table 1).

**Table 1:** Domains and Items of the AGREE II

Domain 1. Scope and Purpose	
Item 1. The overall objective(s) of the guideline is (are) specifically described.	
Item 2. The health question(s) covered by the guideline is (are) specifically described.	
Item 3. The population (patients, public, etc.) to whom the guideline is meant to apply is specifically described.	
Domain 2. Stakeholder Involvement	
Item 4. The guideline development group includes individuals from all relevant professional groups.	
Item 5. The views and preferences of the target population (patients, public, etc.) have been sought.	
Item 6. The target users of the guideline are clearly defined.	
Domain 3. Rigour of Development	
Item 7. Systematic methods were used to search for evidence.	
Item 8. The criteria for selecting the evidence are clearly described.	
Item 9. The strengths and limitations of the body of evidence are clearly described.	
Item 10. The methods for formulating the recommendations are clearly described.	
Item 11. The health benefits, side effects, and risks have been considered in formulating the recommendations.	
Item 12. There is an explicit link between the recommendations and the supporting evidence.	
Item 13. The guideline has been externally reviewed by experts prior to its publication.	
Item 14. A procedure for updating the guideline is provided.	
Domain 4. Clarity of Presentation	
Item 15. The recommendations are specific and unambiguous.	
Item 16. The different options for management of the condition or health issue are clearly presented.	
Item 17. Key recommendations are easily identifiable.	
Domain 5. Applicability	
Item 18. The guideline describes facilitators and barriers to its application.	
Item 19. The guideline provides advice and/or tools on how the recommendations can be put into practice.	
Item 20. The potential resource implications of applying the recommendations have been considered.	
Item 21. The guideline presents monitoring and/or auditing criteria.	
Domain 6. Editorial Independence	
Item 22. The views of the funding body have not influenced the content of the guideline.	
Item 23. Competing interests of guideline development group members have been recorded and addressed.	
Overall Guideline Assessment	
1. Rate the overall quality of this guideline.	
2. I would recommend this guideline for use.	

*Abbreviations:* AGREE, Appraisal of Guidelines for Research and Evaluation

Previous studies regarding the quality of CPGs were limited to specific health topics or regions [21–39] and systematic reviews using these studies [40–42]. Regarding the relationship between quality and application of CPGs, O’Sullivan et al. clarified that high “scores in some domains of AGREE II tool were significantly associated with reductions in nonadherent testing” [32].

The AGREE II overall assessment indicates the general quality of CPGs. The user manual states that the “overall assessment requires the user to make a judgment as to the quality of the guideline, taking into account the criteria considered in the assessment process” [19]. Therefore, AGREE II items and domains can affect the overall assessment. Although several studies have revealed the correlation between domain scores and the overall assessment, they did not adjust the influence between domains [30, 39, 40]. Adjusting such influence, Hoffman-Eßer et al. demonstrated the influence of domains on the overall assessment [42]. The influence of items has been only indicated in a questionnaire survey asking the corresponding authors of CPG evaluation studies to rate the strength of items in the overall assessment [43]. However, the influence of items on the overall assessment has not been examined using the results of CPGs evaluation.

Clarifying the items that have a strong influence on the overall assessment of CPGs will enable CPG developers to recognize the items they should focus on in the process of CPG development. Additionally, it will suggest items to be focused in the CPG evaluation process. Based on the results of evaluation using AGREE II, this study aims to investigate the influence of AGREE II items on the overall assessment of CPGs.

## Methods

### Clinical practice guidelines selection and evaluation

Medical librarians at Toho University Medical Media Center, which has managed a Japanese guidelines clearinghouse since 2001, collected CPGs published in Japan from 2011 to 2015. CPGs were selected based on the following criteria: (1) the title includes the terms “guideline,” “guidance,” or “guide,” (2) the methodology describes the CPG development process based on existing evidence, and (3) the theme relates to clinical practice and not to topics such as medical ethics and animal experimentation. CPGs whose target readers were patients were excluded from this study.

Three appraisers, consisting of experienced medical librarians and CPG researchers, independently evaluated these selected CPGs using AGREE II, which is composed of 23 items grouped into 6 domains and 2 overall assessment items and rated on a 7-point scale (“Strongly Disagree” to “Strongly Agree”). One of the overall assessment items is to rate the quality of the overall CPG on 7-point scale (“Lowest possible quality” to “Highest possible quality”), and the other is to decide whether the CPG would be recommended for use in practice [19].

### Calculating scores

The mean values of the item assessment by the three appraisers were adopted as item scores (1 to 7). According to the “User Manual,” domain scores were “calculated by summing up all the scores of individual items in a domain and by scaling the total as a percentage of maximum possible score for that domain” [19]; these ranged from 0 to 100.

The first overall assessment item is the overall quality rating item, “Rate the overall quality of this guideline” and the second is the CPG endorsement item, “I would recommend this guideline for use.” Users are required to judge the quality of the CPGs and are “also asked whether he/she would recommend use the guideline” [19]. This study used the first overall assessment item as it was more directly related to the methodological quality of CPGs. The mean value of the three appraisers’ rating of the overall quality item was calculated (1 to 7).

### Data analysis

We calculated the intraclass coefficient (ICC) with its 95% confidence interval (95% CI) as an indicator of overall agreement between the three appraisers. A degree of agreement of < 0.00 is poor, between 0.01 and 0.20 is slight, from 0.21 to 0.40 is fair, from 0.41 to 0.60 is moderate, from 0.61 to 0.80 is substantial, and from 0.81 to 1.00 is almost perfect [44].

The influence of the 6 domain scores (independent variables) on the overall assessment score (dependent variable) was examined using a multiple linear regression model. Subsequently, the influence of the 23 item scores (independent variables) on the overall assessment score (dependent variable) was examined using a stratified multiple linear regression model. All 23 item scores were used for Model 1 and the item scores with significant influence were used for Model 2. The CPG publication years were used for adjustment in these analyses.

The data were analyzed using SPSS Statistics version 25, and a *P* value < 0.05 was considered statistically significant.

## Results

### Included clinical practice guidelines

**Fig. 1:**
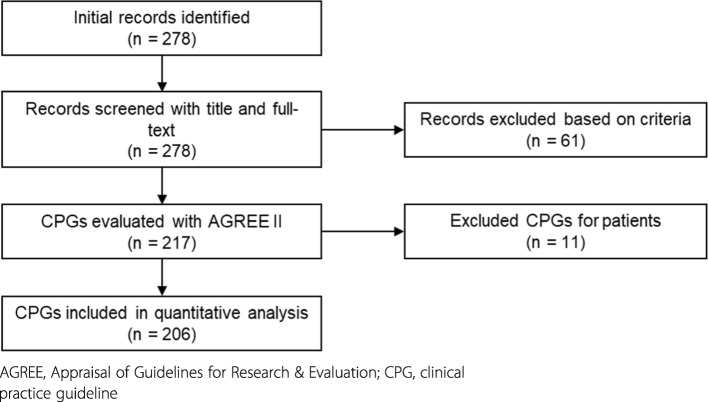
Clinical practice guidelines selection flowchart. Abbreviations:

A total of 278 CPGs were published from 2011 to 2015. Among them, 61 were excluded based on the criteria and a further 11 CPGs for patients were not used. The remaining 206 CPGs were used for the analysis (Additional file 1). Figure 1 shows the flowchart of CPGs retrieved in this study. The number of CPGs was found to have increased; 28 (13.6%) were published in 2011, 34 (16.5%) in 2012, 48 (23.3%) in 2013, 41 (19.9%) in 2014, and 55 (26.7%) in 2015. Academic organizations developed 169 CPGs (82.0%), research groups funded by the Japanese Ministry of Health, Labour and Welfare developed 29 CPGs (14.1%), and other organizations developed 7 CPGs (3.4%). Eighty-three CPGs (40.3%) were revised versions.

### AGREE II scores

The ICC was 0.758 (95% CI: 0.746–0.770), suggesting that there was substantial agreement among the three appraisers.

Table 2 shows mean domain scores, mean overall assessment score, and mean item scores with standard deviations for all CPGs. Mean domain scores were higher in Domain 1 (87.3) and Domain 4 (81.1) than in the other domains (60.7 in Domain 2, 58.8 in Domain 3, 47.4 in Domain 5, and 55.4 in Domain 6). Large standard deviations were observed in Domain 3 (23.1) and Domain 6 (30.1).

**Table 2:** Mean (SD) AGREE II domain, overall, and item scores (*n* = 206)

Domain 1	87.3	(11.1)	
Item 1		6.3	(0.9)
Item 2		6.2	(0.7)
Item 3		6.2	(0.7)
Domain 2	60.7	(13.3)	
Item 4		4.8	(0.7)
Item 5		3.3	(1.3)
Item 6		5.8	(1.2)
Domain 3	58.8	(23.1)	
Item 7		4.0	(2.3)
Item 8		4.4	(1.7)
Item 9		4.3	(1.5)
Item 10		4.5	(1.7)
Item 11		5.5	(1.1)
Item 12		5.5	(1.4)
Item 13		3.4	(2.0)
Item 14		4.5	(2.3)
Domain 4	81.1	(13.6)	
Item 15		6.0	(0.8)
Item 16		5.9	(0.9)
Item 17		5.6	(1.2)
Domain 5	47.7	(14.5)	
Item 18		3.9	(1.4)
Item 19		3.7	(1.3)
Item 20		3.7	(1.4)
Item 21		4.1	(0.9)
Domain 6	55.4	(30.1)	
Item 22		5.2	(2.3)
Item 23		3.4	(2.1)
Overall		5.1	(0.7)

*Abbreviations: AGREE* Appraisal of Guidelines, Research and Evaluation, *SD* standard deviation

The mean overall assessment score was 5.1 and its standard deviation was small. The median of the 23 mean item scores was 4.5, mean item scores of Items 5, 13, 19, and 20 were smaller than the 1st quartile of the 23 mean item scores (3.9). The highest mean item score was 6.3 for Item 1, followed by Item 2 (6.2) and Item 3 (6.2), which were from Domain 1. Items in Domain 4 also have high mean item scores (5.6 to 6.0). Standard deviations were also large in items constituting Domain 3 and Domain 6.

### Correlation between domains or items and the overall assessment

Table 3 includes correlation coefficients between domains and the overall assessment, and between items and the overall assessment. Correlation coefficients for the overall assessment were strong in Domain 3 (0.720), moderate in Domain 4 (0.676), Domain 2 (0.566), and Domain 1 (0.509), and weak in Domain 6 (0.409) and Domain 5 (0.404). Except for Item 21, the other items were significantly correlated with the overall assessment. Specifically, items in Domain 3 and Domain 4 had high correlation to the overall assessment. The highest coefficient was observed in Item 10 (*r* = 0.706), followed by Item 8 (*r* = 0.705), Item 12 (*r* = 0.680), and Item 11 (*r* = 0.678).

**Table 3:** Correlation coefficients between overall assessment and domains / items (*n* = 206)

	*r*	*P*
Domain 1	0.509	< 0.001
Item 1	0.402	< 0.001
Item 2	0.486	< 0.001
Item 3	0.438	< 0.001
Domain 2	0.566	< 0.001
Item 4	0.567	< 0.001
Item 5	0.377	< 0.001
Item 6	0.387	< 0.001
Domain 3	0.720	< 0.001
Item 7	0.478	< 0.001
Item 8	0.705	< 0.001
Item 9	0.647	< 0.001
Item 10	0.706	< 0.001
Item 11	0.678	< 0.001
Item 12	0.680	< 0.001
Item 13	0.474	< 0.001
Item 14	0.432	< 0.001
Domain 4	0.676	< 0.001
Item 15	0.651	< 0.001
Item 16	0.497	< 0.001
Item 17	0.571	< 0.001
Domain 5	0.404	< 0.001
Item 18	0.266	< 0.001
Item 19	0.470	< 0.001
Item 20	0.288	< 0.001
Item 21	0.025	0.724
Domain 6	0.409	< 0.001
Item 22	0.318	< 0.001
Item 23	0.360	< 0.001

*Abbreviations:* r, correlation coefficients

There was a difference between the items composing one domain. In particular, the correlation coefficients between items and the overall assessment were found to have large ranges in Domain 2 (0.377 to 0.567), Domain 3 (0.432 to 0.706), and Domain 5 (0.025 to 0.470).

### Influence of six domains on the overall assessment

Domain 3 had the strongest influence on the overall assessment (*β* = 0.469; *P* <  0.001), followed by Domain 4 (*β* = 0.188; *P* = 0.002), Domain 5 (*β* = 0.158; *P* = 0.001), and Domain 6 (*β* = 0.123; *P* = 0.009). Domain 1 and Domain 2 did not have a significant influence. Adjusted R-squared was 0.719 (Table 4).

**Table 4:** Influence of AGREE II six domains on overall assessment (*n* = 206)

	*B*	*SE*	*β*	*P*
Domain 1	−0.016	0.061	−0.015	0.789
Domain 2	0.021	0.052	0.023	0.690
Domain 3	0.247	0.033	0.469	0.000
Domain 4	0.168	0.053	0.188	0.002
Domain 5	0.133	0.040	0.158	0.001
Domain 6	0.050	0.019	0.123	0.009
Adj-R^2^	0.719			

*Abbreviations: AGREE* Appraisal of Guidelines, Research and Evaluation, *B* unstandardized regression coefficient, *SE* standard error, *β* standardized regression coefficient, *Adj-R*^*2*^ adjusted R-squared

This analysis was from regression model that was controlled for publication years

### Influence of 23 items on the overall assessment

Table 5 shows the result of the multiple regression analysis for the influence of 23 items on the overall assessment. In Model 1, which includes all items for analysis, four items showed statistically significant influence on the overall assessment; Item 15 had the strongest influence (*β* = 0.218; *P* = 0.001) followed by Item 8 (*β* = 0.211; *P* = 0.024), Item 19 (*β* = 0.161; *P* = 0.001), and Item 22 (*β* = 0.099; *P* = 0.016). These four items were extracted one by one from Domain 3 (Rigour of Development), Domain 4 (Clarity of Presentation), Domain 5 (Applicability), and Domain 6 (Editorial Independence), which had a significant influence on the overall assessment. Adjusted R-squared was 0.743.

**Table 5:** Influence of AGREE II 23 items on overall assessment (*n* = 206)

	*B*	*SE*	*β*	*P*
Item 1	0.096	0.719	0.007	0.894
Item 2	−0.549	1.126	−0.031	0.626
Item 3	0.257	0.930	0.016	0.782
Item 4	1.289	0.890	0.074	0.150
Item 5	0.262	0.425	0.028	0.538
Item 6	− 0.565	0.518	− 0.057	0.276
Item 7	−0.385	0.362	−0.071	0.288
Item 8	1.530	0.674	0.211	0.024
Item 9	1.206	0.686	0.149	0.081
Item 10	0.765	0.755	0.104	0.312
Item 11	0.198	0.783	0.018	0.800
Item 12	0.139	0.691	0.015	0.841
Item 13	0.576	0.316	0.093	0.070
Item 14	0.389	0.263	0.074	0.141
Item 15	3.302	0.997	0.218	0.001
Item 16	0.704	0.796	0.051	0.378
Item 17	−0.499	0.623	−0.050	0.424
Item 18	0.149	0.490	0.017	0.761
Item 19	1.515	0.437	0.161	0.001
Item 20	−0.124	0.435	− 0.014	0.776
Item 21	−1.052	0.642	−0.074	0.103
Item 22	0.521	0.214	0.099	0.016
Item 23	0.473	0.286	0.080	0.100
Adj-R^2^	0.743			

*Abbreviations: AGREE* Appraisal of Guidelines, Research and Evaluation, *B* unstandardized regression coefficient, *SE* standard error, *β* standardized regression coefficient, *Adj-R*^*2*^ adjusted R-squared

This analysis was from regression model that was controlled for publication years

In Model 2 assesses the influence of these four items, all of which had a significant influence on the overall assessment; Item 8 had the strongest influence (*β* = 0.456; *P* <  0.001) followed by Item 15 (*β* = 0.243; *P* <  0.001), Item 19 (*β* = 0.207; *P* <  0.001), and Item 22 (*β* = 0.173; *P* <  0.001). Adjusted R-squared of Model 2 was 0.721, which was higher than the result of analysis for the influence of domains on the overall assessment, and comparable to the result of Model 1 (Table 6).

**Table 6:** Influence of AGREE II four items on overall assessment (*n* = 206)

	*B*	*SE*	*β*	*P*
Item 8	3.313	0.331	0.456	< 0.001
Item 15	3.679	0.762	0.243	< 0.001
Item 19	1.948	0.376	0.207	< 0.001
Item 22	0.911	0.204	0.173	< 0.001
Adj-R^2^	0.721			

*Abbreviations: AGREE* Appraisal of Guidelines, Research and Evaluation, *B* unstandardized regression coefficient, *SE* standard error, *β* standardized regression coefficient, *Adj-R*^*2*^ adjusted R-squared

This analysis was from regression model that was controlled for publication years

## Discussion

Based on the evaluation results of 206 CPGs using AGREE II, this study examined the influence of 23 items on the overall assessment of CPGs using regression analyses.

Domain scores were found to be higher in Domain 1 (Scope and Purpose) and Domain 4 (Clarity of Presentation) than those in the other domains. Two previous systematic reviews of CPGs reported the same tendency [40, 41]. These results might suggest that there was room for improvement in Domain 2 (Stakeholder Involvement), Domain 3 (Rigour of Development), Domain 5 (Applicability), and Domain 6 (Editorial Independence).

Domain 3 (Rigour of Development), Domain 4 (Clarity of Presentation), Domain 5 (Applicability), and Domain 6 (Editorial Independence) were found to have a significant influence on the overall assessment. Domain 3 had the strongest among the 6 domains. Analyzing the results of evaluation of CPGs published from 1992 to 2015, Hoffmann-Eßer et al. reported that all domains had a significant influence on the overall assessment, and Domain 3 had the strongest influence [42]. In this study, no relationship was observed between the overall assessment and Domain 1 or Domain 2, and relatively small standard deviations of Domain 1 and Domain 2 reflecting homogeneity among CPGs may explain the lack of a relationship. Although the scores of Domain 1 are high, low scores of Domain 2 may suggest that a method to improve stakeholder involvement should be developed.

A significant influence on the overall assessment was observed in Item 8 (The criteria for selecting the evidence are clearly described.), Item 15 (The recommendations are specific and unambiguous.), Item 19 (The guideline provides advice and/or tools on how the recommendations can be put into practice.), and Item 22 (The views of the funding body have not influenced the content of the guideline.). Item 8 and Item 22 are related to the trustworthiness of CPGs, Item 15 and Item 19 are related to the implementation of CPGs. These four items explained a large proportion of the variance in the overall assessment. AGREE II item scores suggest that effective detailed notes as well as domain scores for appraising the quality of CPGs should be provided. CPG developers could improve the quality of CPGs by focusing on these four items.

While detailed CPG evaluation tools have been prepared for CPG developers [45–47], complex assessment tools with many items was not applicable in busy clinical settings. The AGREE II user manual suggested that users should first carefully read the guideline document in full before applying the AGREE II, and attempt to identify all information about the guideline development process in addition to the guideline document [19]. However, it is difficult for CPGs appraisers in busy settings. Consequently, some rapid assessment tools were developed such as the AGREE Global Rating Scale with four items [48], the rapid-assessment Mini-Checklist (MiChe) tool with eight items [49], and the iCAHE Guideline Quality Checklist with 14 items [50]. They were verified by comparing to the results of CPG assessment with AGREE II. This study clarified that four AGREE II items had a significant influence on the overall assessment, and they can explain 72.1% of the variance. These four items may constitute a CPG rapid assessment tool.

This study examined the quality of CPGs using AGREE II, which is a tool for assessing the quality of CPGs in terms of the methodological rigour and transparency [19]. However, health care providers consider not only methodological quality but also the content of CPGs before they apply recommendations suggested in CPGs for their daily practice. Additionally, it was suggested that the quality of CPG development did not have a direct link to the validity of CPG content [51, 52]. Therefore, to assure the time for assessing both methodological quality and content validity of CPGs in clinical practice, there is a need for rapid assessment tools for methodological quality of CPGs, as previous studies and this study have shown. Until the validity of our very short list of 4 items confirmed, health-care professionals can at least use the shorter checklists referred above [49–51].

Ours is a pioneering study, which is based on a moderate sample size with substantial agreement among appraisers, that assess the influence of the items on the overall assessment. This study has the following limitations. 1) Although we analyzed 206 CPGs published from 2011 to 2015, the number of CPGs was still insufficient in Model 1. 2) We did not consider the relationship between 23 items and the CPG endorsement item. In future, it is necessary to use a sufficient number of CPGs, improve accuracy, and to investigate the influences of domains and items on overall recommendation assessment. 3) The samples examined in the present study were limited to CPGs developed by academic organizations, research groups, and other organizations in Japan. While this study showed that domain scores were similar to the systematic reviews conducted in other countries, the results of our study should be applied to other regions with caution.

## Conclusion

This study showed that Domain 3 (Rigour of Development), Domain 4 (Clarity of Presentation), Domain 5 (Applicability), and Domain 6 (Editorial Independence) had influence on the overall assessment. It was also revealed that Item 8 (The criteria for selecting the evidence are clearly described.), Item 15 (The recommendations are specific and unambiguous.), Item 19 (The guideline provides advice and/or tools on how the recommendations can be put into practice.), and Item 22 (The views of the funding body have not influenced the content of the guideline.) significantly influenced the overall assessment and these four items could explain 72.1% of the variance. Specifically, they present the key points on the quality of methodology, not contents, that CPG developers should focus on in the development process, and that CPG appraisers should focus on in the evaluation of CPGs.

## Supplementary information

**Additional file 1.** Included clinical practice guidelines. This file shows included clinical practice guidelines for this analysis (*n* = 206).

## Data Availability

The datasets used and analyzed during the current study are available from the corresponding author on reasonable request.

## Abbreviations

AGREE: Appraisal of Guidelines for Research and Evaluation
CPG: Clinical Practice Guideline
PRISMA: Preferred Reporting Items for Systematic Reviews and Meta-Analyses
