# Supplementary material for: The structure of the quality of clinical practice guidelines with the items and overall assessment in AGREE II: a regression analysis
Source: BMC Health Serv Res. 2019 Nov 4;19:788. doi: 10.1186/s12913-019-4532-0 (PMC6827207; doi:10.1186/s12913-019-4532-0)
Supplement: Supplementary file 1 — Additional file 1. Included clinical practice guidelines. This file shows included clinical practice guidelines for this analysis (n = 206). [file 12913_2019_4532_MOESM1_ESM.docx]

**Additional file 1** Included clinical practice guidelines

All bibliographic information of CPGs is available from the website (https://guideline.jamas.or.jp/) managed by Toho University and Japan Medical Abstracts Society. Unfortunately, there is no database of English version of Japanese CPGs.

[1] Japan Resuscitation Council, Japan Emergency Care Foundation. Clinical Practice Guideline for Resuscitation. 2011.

[2] Research Committee for Mixed Connective Tissue Disease of the Ministry of Health, Labour and Welfare, Japan. Clinical Practice Guideline for Mixed Connective Tissue Disease. 2011.

[3] Research Committee for ANCA-associated Vasculitis, Intractable Angiitis and Progressive Renal Disorder of the Ministry of Health, Labour and Welfare, Japan. Clinical Practice Guideline for ANCA-associated Vasculitis. 2011.

[4] The Japanese Respiratory Society. Clinical Practice Guideline for Healthcare Associated Pneumonia. 2011.

[5] Research Committee on Intractable Inflammatory Bowel Disorders of the Ministry of Health, Labour and Welfare of Japan, The Japanese Society of Gastroenterology. Clinical Practice Guideline for Crohn Disease. 2011.

[6] Research Committee on Intractable Hepatic and Biliary Tract Disease of the Ministry of Health, Labour and Welfare of Japan . Clinical Practice Guideline for Primary Biliary Cirrhosi. 2011.

[7] Japan Thyroid Association. Guidelines for the Treatment of Graves' Disease in Japan. 2011.

[8] Japan College of Rheumatology. Clinical Practice Guideline for Management of Rheumatoid Arthritis. 2011.

[9] Research Committee on QOL of Specified Disease Patient of the Ministry of Health, Labour and Welfare of Japan. Clinical Practice Guideline for Home Music Therapy of Amyotrophic Lateral Sclerosis (ALS). 2011.

[10] The Japanese Society of NPH. Clinical Practice Guideline for Diagnosis and Management of Fetal Hydrocephalus. 2011.

[11] Japan Society of Facial Nerve Research. Clinical Practice Guideline for Facial Palsy; Bell's palsy and Hunt syndrome. 2011.

[12] Japanese Society of Neurology. Evidence-based Clinical Practice Guidelins for Parkinson's Disease. 2011.

[13] The Japan Diabetes Society, The Japanese Society for Pediatric Endocrinology. Clinical Practice Guideline for Management of Diabetes in Children and Puberty. 2011.

[14] Japanese Society of Pediatric Oncology. Clinical Practice Guideline for Pediatric Oncology. 2011.

[15] Japan Society of Pain Clinicians. Clinical Practice Guideline for Pharmacotherapy of Neuropathic Pain. 2011.

[16] Japanese Society for Clinical Renal Transplantation. Clinical Practice Guideline for Internal Medicine and Pediatric Complication after Kidney Transplantation. 2011.

[17] Japanese Society for Palliative Medicine. Clinical Guidelines for Gastrointestinal Symptoms in Cancer Patients. 2011.

[18] Japanese Society for Palliative Medicine. Clinical Guidelines for Respiratory Symptoms In Cancer Patients. 2011.

[19] Japanese Breast Cancer Society. Evidence-based Clinical Practice Guidelines for Breast Cancer 1, Treatment. 2011.

[20] Japanese Breast Cancer Society. Evidence-based Clinical Practice Guidelines for Breast Cancer 2, Epidemiology and Diagnosis. 2011.

[21] The Japanese Orthopaedic Association. Clinical Practice Guideline for Ossification of Posterior Longitudinal Ligament of the Cervical Spine. 2011.

[22] The Japanese Orthopaedic Association. Clinical Practice Guideline for Lumbar Spinal Stenosis . 2011.

[23] The Japanese Orthopaedic Association. Clinical Practice Guideline for Lumbar Disc Herniation. 2011.

[24] The Japanese Orthopaedic Association. Clinical Practice Guideline for Femoral Neck Fracture and Intertrochanteric Femoral Fracture. 2011.

[25] The Japanese Urological Association. Evidence-based Clinical Practice Guidelines for Benign Prostatic Hyperplasia. 2011.

[26] Research Committee on Behcet’s Disease of the Ministry of Health, Labour and Welfare of Japan. Clinical Practice Guideline for Behcet's Disease　. 2011.

[27] Research Committee on Vestibular Dysfunction of the Ministry of Health, Labour and Welfare of Japan. Clinical Practice Guideline for Ménierè Disease. 2011.

[28] Japanese Society of Periodontology. Clinical Practice Guideline for Antimicrobial Treatment of Periodontal Disease Patient. 2011.

[29] The Japanese Association for The Surgery of Trauma, Japanese Association for Acute Medicine. Clinical Practice Guideline for advanced trauma, Japan Advanced Trauma Evaluation and Care; JATEC. 2012.

[30] Japan Society for Eating Disorders. Clinical Practice Guideline for Management of Eating Disorder. 2012.

[31] Research Committee on Von Hippel–Lindau Disease of the Ministry of Health, Labour and Welfare of Japan. Clinical Practice Guideline for Von Hippel-Lindau (VHL) Disease. 2012.

[32] Research Committee on Fall Prevention in the Elderly of the Ministry of Health, Labour and Welfare of Japan. Guidelines for Fall Prevention in the Elderly. 2012.

[33] The Japanese Circulation Society. Clinical Practice Guideline for Dilated Cardiomyopathy and Related Secondary Cardiomyopathy. 2012.

[34] Japan Atherosclerosis Society. Japan Atherosclerosis Society (JAS) Guidelines for Prevention of Atherosclerotic Cardiovascular Diseases. 2012.

[35] Research Committee on Primary Lymphedema of the Ministry of Health, Labour and Welfare of Japan. Clinical Practice Guideline for Diagnosis of Primary Lymphedema. 2012.

[36] The Japanese Respiratory Society. Clinical Practice Guideline for Cough. 2012.

[37] Japanese Study Group on Pancreatico biliary Maljunction, Japan Biliary Association. Clinical Practice Guideline for Pancreaticobiliary Maljunction. 2012.

[38] Japanese Association for Emergency Psychiatry. Clinical Practice Guideline for Psychiatric Emergency Care. 2012.

[39] The Japanese Society for Tuberculosis. Clinical Practice Guideline for Tuberculosis. 2012.

[40] Japanese Society of Pediatric Allergy and Clinical Immunology. Japanese Pediatric Guideline for Food Allergy. 2012.

[41] Japanese Society of Emergency Pediatrics. Evidence-based Guidelines for the Management of Intussusception in Children. 2012.

[42] Japanese Society for Clinical Renal Transplantation. Clinical Practice Guideline for Cytomegalovirus Infection after Kidney Transplantation. 2012.

[43] The Japanese Dermatological Association. Clinical Practice Guideline for Wound and Burn. 2012.

[44] Japanese Society of Medical Oncology. Clinical Practice Guideline for Febrile Neutropenia. 2012.

[45] Japanese Society of Myeloma. Clinical Practice Guideline for Multiple Myeloma. 2012.

[46] The Japan Esophageal Society. Guidelines for Diagnosis and Treatment of Carcinoma of the Esophagus. 2012.

[47] Japanese Society for Cancer of the Colon and Rectum. JSCCR Guidelines for the Clinical Practice of Hereditary Colorectal Cancer. 2012.

[48] The Japanese Urological Association. Clinical Practice Guideline for Prostate Cancer. 2012.

[49] The Japanese Orthopaedic Association. Clinical Practice Guideline for Diagnosis of Soft-Tissue Tumor. 2012.

[50] The Japanese Orthopaedic Association. Clinical Practice Guideline for Distal Radius Fracture. 2012.

[51] Japan Osteoporosis Society, The Japanese Society for Bone and Mineral Research, Japan Osteoporosis Foundation. Clinical Practice Guideline for Prevention and Management of Osteoporosis. 2012.

[52] The Japanese Orthopaedic Association. Clinical Practice Guideline for Low-Back Pain. 2012.

[53] The Japanese Orthopaedic Association. Clinical Practice Guideline for Anterior Cruciate ligament (ACL) Injuries . 2012.

[54] Japanese Society of Allergology. Guidelines for the Management of Atopic Dermatitis. 2012.

[55] Research Committee on Refractory Nephrotic Syndrome of the Ministry of Health, Labour and Welfare of Japan. Clinical Practice Guideline for Nephrotic Syndrome. 2012.

[56] Japanese Society of Nephrology, Japan Radiological Society, The Japanese Circulation Society. Clinical Practice Guideline for Iodinated Contrast Media in Renal Impairment Patient. 2012.

[57] The Japanese Continence Society, Japan Medical Society of Spinal Cord Lesion. Clinical Practice Guideline for Dysuria of Spinal Cord Injury. 2012.

[58] The Japanese Society for Sexual Medicine. Clinical Practice Guideline for Erectile Dysfunction (ED). 2012.

[59] Japanese Society of Ocstetrics and Gynecology, Japan Society for Menopause and Women's Health. Hormone Replacement Therapy Guideline. 2012.

[60] The Japanese Neuro-ophthalmology Society. Clinical Practice Guideline for Blepharospasm. 2012.

[61] The Oto-Rhino-Laryngological Society of Japan. Clinical Practice Guideline for　Dysphagia in Otorhinolaryngology Outpatient Department. 2012.

[62] Japan Infection Prevention and Control Conference for National and Public University Hospitals. Clinical Practice Guideline for Management of Hospital Infection. 2012.

[63] Japanese Society for Parenteral & Enteral Nutrition. Clinical Practice Guideline for Parenteral and Enteral Nutrition. 2013.

[64] The Japan Diabetes Society. Evidence-based Practice Guideline for the Treatment of Diabetes in Japan. 2013.

[65] Clinical Practice Guideline Development Group for the　Pompe Disease. Clinical Practice Guideline for Diagnosis and Management of Pompe disease (glycogenosis type II). 2013.

[66] Committee for Japanese Guideline for Diagnosis and Management of Occupational Allergic Disease. Japanese Guidelines for Occupational Allergic Diseases. 2013.

[67] Japanese Society of Latex Allergy. Clinical Practice Guideline for Safety of Latex Allergy. 2013.

[68] The Japanese Circulation Society. Clinical Practice Guideline for Pharmacotherapy of Heart Disease in Children. 2013.

[69] Research Committee on Immunity and Allergy of the Ministry of Health, Labour and Welfare of Japan. Clinical Practice Guideline for Prevention and Management of Asthma. 2013.

[70] The Japanese Respiratory Society. Clinical Practice Guideline for Diagnosis and Treatment of Chronic Obstructive Pulmonary Disease (COPD). 2013.

[71] Japanese Society for Abdominal Emergency Medicine, Japanese Society of Hepato-Biliary-Pancreatic Surgery, Japan Biliary Association, Japan Society for Surgical Infection, Japan Radiological Society. Clinical Practice Guideline for Acute Cholangitis and Acute Cholecystitis. 2013.

[72] Japan College of Fibromyalgia Investigation. Clinical Practice Guideline for Fibromyalgia. 2013.

[73] Japanese Society of Neurology, The Japanese Headache Society. Clinical Practice Guideline for　Chronic Headache. 2013.

[74] Japanese Society of Neurology. Practical Guideline for Guillain-Barré Syndrome and Fisher Syndrome. 2013.

[75] Japanese Society of Neurology. Clinical Practice Guideline for Chronic Inflammatory Demyelinating Polyradiculoneuropathy and Multifocal Motor Neuropathy. 2013.

[76] Japanese Society of Neurology. Clinical Practice Guideline for Amyotrophic Lateral Sclerosis. 2013.

[77] Research Committee on Prion Disease and Slow Virus Infection of the Ministry of Health, Labour and Welfare of Japan. Clinical Practice Guideline for Progressive Multifocal Leukoencephalopathy (PML). 2013.

[78] Japanese Society of Mood Disorders. Clinical Practice Guideline for Major Depressive Disorder and Bipolar Disorder. 2013.

[79] Japanese Society for Sexually Transmitted Infections. Clinical Practice Guideline for Diagnosis and Management of Sexually Transmitted Disease. 2013.

[80] Japanese Society of Chemotherapy, The Japanese Association for Infectious Diseases. Clinical Practice Guideline for Management of MRSA Infection. 2013.

[81] The Japanese Society of Intensive Care Medicine. Clinical Practice Guideline for Sepsis. 2013.

[82] The Japanese Society for Pediatric Gastroenterology, Hepatology and Nutrition, Japanese Society for Pediatric Neurogastroenterology. Clinical Practice Guideline for Chronic Functional Constipation in Children. 2013.

[83] The Japanese Society for Pediatric Nephrology. Clinical Practice Guideline for Infantile Idiopathic Nephrotic Syndrome. 2013.

[84] Japan Society of Pain Clinicians. Clinical Practice Guideline for Management of Pain Clinic. 2013.

[85] Japanese Society for Palliative Medicine. Clinical Practice Guideline for Fluid Therapy of Terminal Cancer Patient. 2013.

[86] Japanese Association of Rehabilitation Medicine. Clinical Practice Guideline for Rehabilitation of Cancer. 2013.

[87] Japanese Society of Medical Oncology. Clinical Practice Guideline for Tumour Lysis Syndrome (TLS). 2013.

[88] Japan Society of Clinical Oncology. Clinical Practice Guideline for Proper Use of Granular Colony Stimulating Factor (G-CSF). 2013.

[89] Japanese Breast Cancer Society. Evidence-based Clinical Practice Guidelines for Breast Cancer, Treatment. 2013.

[90] Japanese Breast Cancer Society. Evidence-based Clinical Practice Guidelines for Breast Cancer, Epidemiology and Diagnosis. 2013.

[91] Japan Pancreas Society. Evidence-based Clinical Practice Guidelines for Pancreatic Cancer. 2013.

[92] Japan Thyroid Association. Clinical Practice Guideline for Thyroid Nodule. 2013.

[93] Clinical Practice Guideline Development Group for the Multiple Endocrine Neoplasia. Clinical Practice Guideline for Multiple Endocrine Neoplasia. 2013.

[94] The Japan Society of Gynecologic Oncology. Clinical Practice Guideline for　Uterine Cancer. 2013.

[95] Japan Society for Head and Neck Cancer. Clinical Practice Guideline for Head and Neck Cancer. 2013.

[96] Japanese Society of Oral Oncology, Japanese Society of Oral and Maxillofacial Surgeons. Evidence-based Clinical Practice Guidelines for Oral Cancer. 2013.

[97] Clinical Practice Guideline Development Group for the Treatment and Management of Severe Head Injury. Clinical Practice Guideline for Treatment and Management of Severe Head Injury. 2013.

[98] Japan Society for Stereotactica and Functional Neurosurgery. Clinical Practice Guideline for Management of Stereotactical and Functional Neurosurgery. 2013.

[99] Japanese Society of Awake Surgery. Clinical Practice Guideline for Awake Surgery. 2013.

[100] The Japanese Urological Association,Japanese Society of Endourology and ESWL, Japanese Society on Urolithiasis Research. Clinical Practice Guideline for Urolithiasis. 2013.

[101] Japanese Society of Nephrology. Evidence-based Clinical Practice Guideline for CKD. 2013.

[102] The Japan Society of Gynecologic and Obstetric Endoscopy and Minimally Invasive Therapy. Clinical Practice Guideline for Gynecological Endoscopic Surgery. 2013.

[103] The Japanese Continence Society. Clinical Practice Guideline for Lower Urinary Tract Symptom in Woman. 2013.

[104] Research Committee on Pregnancy and Childbirth of the Ministry of Health, Labour and Welfare of Japan. Clinical Practice Guideline for Pregnancy and Delivery. 2013.

[105] Japan Otological Society, Japan Society for Pediatric ORL, Japan Society for Infectious Diseases in Otolaryngology. Guidelines for Acute Otitis Media in Children. 2013.

[106] Clinical Practice Guideline Development Group for the Management of Allergic Rhinitis. Allergic Rhinitis Guide. 2013.

[107] Clinical Practice Guideline Development Group for the Management of Allergic Rhinitis. Practical Guideline for the Management of Allergic Rhinitis in Japan <PG-MARJ>. 2013.

[108] Japanese Society of Periodontology. Clinical Practice Guideline for Regenerative Treatment of Periodontal Disease Patient. 2013.

[109] Japanese Society of Dentistry for Medically Compromised Patient, Japanese Society of Oral and Maxillofacial Surgeons, Japanese Society of Gerodontology. Clinical Practice Guideline for Tooth Extraction of Antithrombotic Therapy Patient. 2013.

[110] The Japanese Academy of Clinical Periodontology. Clinical Practice Guideline for Implant Treatment of Periodontal Disease Patient. 2013.

[111] Research Committee on Behcet’s Disease of the Ministry of Health, Labour and Welfare of Japan. Clinical Practice Guideline for Neuro-Behcet’s Disease. 2014.

[112] Japanese Society of Allergology. Anaphylaxis Guideline. 2014.

[113] Research Committee on Intractable Angiitis and Progressive Renal Disorder of the Ministry of Health, Labour and Welfare of Japan. Clinical Practice Guideline for ANCA-associated Vasculitis. 2014.

[114] The Japanese Society of Hypertension. Japanese Society of Hypertension Guidelines for the Management of Hypertension (JSH 2014). 2014.

[115] The Japanese Association for Infectious Diseases, Japanese Society of Chemotherapy. Clinical Practice Guideline for Management of Respiratory Infection. 2014.

[116] The Japanese Society of Mycolpasmology. Clinical Practice Guideline for Mycoplasma Pneumonia. 2014.

[117] The Japanese Society of Gastroenterology. Evidence-based Clinical Practice Guidelines for Functional Dyspepsia. 2014.

[118] The Japanese Society of Gastroenterology. Evidence-based Clinical Practice Guidelines for Irritable Bowel Syndrome. 2014.

[119] Research Committee on Intractable Hepatic and Biliary Tract Disease of the Ministry of Health, Labour and Welfare of Japan. Clinical Practice Guideline for Autoimmune Hepatitis （AIH). 2014.

[120] The Japanese Society of Gastroenterology. Clinical Practice Guideline for Non-Slcoholic Fatty Liver Disease (NAFLD) and Non-Alcoholic Steatohepatitis　（NASH）. 2014.

[121] Japan College of Rheumatology. Clinical Practice Guideline for Rheumatoid Arthritis. 2014.

[122] Japanese Society of Neurology, The Japanese Society of Child Neurology, National Center of Neurology and Pcychiatry. Practical Guideline for Duchenne Muscular Dystrophy (DMD). 2014.

[123] Japanese Society of Neurology. Clinical Practice Guideline for Myasthenia Gravis. 2014.

[124] Japanese Association of Rehabilitation Medicine. Clinical Practice Guideline for Pulmonary Rehabilitation of Neuromuscular Disease and Spinal Cord Injury. 2014.

[125] Research Committee on Prion Disease and Slow Virus Infection of the Ministry of Health, Labour and Welfare of Japan. Clinical Practice Guideline for Prion Disease. 2014.

[126] Research Committee on Proper Use of Sleeping Pills of the Ministry of Health, Labour and Welfare of Japan, The Japanese Society of Sleep Research. Clinical Practice Guideline for Proper Use of Hypnotics. 2014.

[127] The Japanese Society for Medical Mycology. Guidelines for Management of Deep-Seated Mycoses. 2014.

[128] Japanese Society of Chemotherapy, The Japanese Association for Infectious Diseases. Clinical Practice Guideline for Management of MRSA Infection. 2014.

[129] Japanese Society of Pediatric Pulmonology. Clinical Practice Guideline for Cough in Children. 2014.

[130] Japan Society of Pain Clinicians. Guidelines for the Interventional Pain Treatment. 2014.

[131] The Japanese Society of Therapeutic Drug Monitoring, The Japan Society for Transplantation. Clinical Practice Guideline for Therapeutic Drug Monitoring (TDM) of Immunosuppressant. 2014.

[132] The Japanese Association for The Surgery of Trauma. Clinical Practice Guideline for Expert Trauma, Japan Expert Trauma Evaluation and Care；JETEC. 2014.

[133] Japan Society of Pain Clinicians. Guidelines for the Interventional treatment of Cancer Pain. 2014.

[134] Japanese Society for Palliative Medicine. Clinical Guidelines for Cancer Pain Management. 2014.

[135] Research Committee on Support System for Fertility Preservation of Breast Cancer Patient of the Ministry of Health, Labour and Welfare of Japan, Japan Society for Fertility Preservation. Clinical Practice Guideline for Pregnancy and Delivery and Reproductive Medicine of Breast Cancer Patient. 2014.

[136] The Japan Lung Cancer Society. Evidence-based Clinical Practice Guidelines for Lung Cancer. 2014.

[137] Japan Society of Clinical Oncolog, Japanese Gastric Cancer Association, Japanese Study Group on GIST. Clinical Practice Guideline for GIST. 2014.

[138] Japanese Gastric Cancer Association. Japanese Gastric Cancer Treatment Guidelines 2014 (ver. 4). 2014.

[139] Japanese Society for Cancer of the Colon and Rectum. JSCCR guidelines ... for the Treatment of Colorectalcancer. 2014.

[140] The Japanese Society of Gastroenterology. Clinical Practice Guideline for　 Colon Polyp. 2014.

[141] Japanese Society of Hepato-Biliary-Pancreatic Surgery. Evidence-based Clinical Practice Guidelines for Biliary Tract Cancer. 2014.

[142] The Japanese Urological Association. Clinical Practice Guideline for Renal Pelvic Cancer and Ureteral Cancer. 2014.

[143] The Japan Society of Coloproctology. Clinical Practice Guideline for Anal Disease. 2014.

"[144] The Japanese Orthopaedic Association. Clinical Practice Guideline for

Hallux Calgus. 2014."

[145] Japanese Association of Rehabilitation Medicine. Clinical Practice Guideline for　Rehabilitation of Cerebral Palsy. 2014.

[146] Research Committee on Progressive Renal Disorder of the Ministry of Health, Labour and Welfare of Japan. Clinical Practice Guideline for Polycystic Kidney Disease (PKD). 2014.

[147] Clinical Practice Guideline Development Group for the Diagnosis and Management of Hemolytic-Uremic Syndrome. Clinical Practice Guideline for Diagnosis and Management of Hemolytic Uremic Syndrome. 2014.

[148] The Japanese Urological Association. Clinical Practice Guideline for Acute Scrotum. 2014.

[149] Japanese Society of Ocstetrics and Gynecology, Japan Association of Obstetricians and Gynecologists. Guideline for Gynecological Practice in Japan 2014. 2014.

[150] Japanese Society of Ocstetrics and Gynecology, Japan Association of Obstetricians and Gynecologists. Guideline for Obstetrical Practice in Japan 2014. 2014.

[151] Japanese Society of Periodontology. Clinical Practice Guideline for Regenerative Treatment of Periodontal Disease Patient. 2014.

[152] Japan Radiological Society,　Research Committee on Imaging Interpretation after Death of the Ministry of Health, Labour and Welfare of Japan . Clinical Practice Guideline for Imaging Interpretation after Death. 2015.

[153] The Japanese Respiratory Society. Clinical Practice Guideline for Noninvasive positive pressure ventilation therapy. 2015.

[154] The Japanese Society of Intensive Care Medicine. Japanese guidelines for the management of pain, agitation, and delirium in intensive care unit (J-PAD). 2015.

[155] The Japan Geriatrics Society. Guidelines for medical treatment and its safety in the elderly. 2015.

[156] Clinical Practice Guideline Development Group for the Privention and Management of Athma. Clinical Practice Guideline for Prevention and Management of Asthma. 2015.

[157] Japanse Society fo Abdominal Emergency Medicine, Japan Primary Care Association, Japan Radiological Society, Japanese Society of Ocstetrics and Gynecology, Japanese Society for Vascular Surgery. Clinical Practice Guideline for Acute Abdomen. 2015.

[158] The Japanese Society of Gastroenterology. Evidence-based clinical practice guidelines for peptic ulcer. 2015.

[159] The Japanese Society of Gastroenterology. Evidence-based clinical practice guidelines for GERD. 2015.

[160] Japanse Society fo Abdominal Emergency Medicine, Research Committee on Refractory Pancreatic Disease of the Ministry of Health, Labour and Welfare of Japan, Japanese Society of Hepato-Biliary-Pancreatic Surgery, Japan Pancreas Society, Japan Radiological Society. Clinical Practice Guideline for Acute Pancreatitis. 2015.

[161] The Japanese Society of Gastroenterology. Clinical Practice Guideline for chronic pancreatitis. 2015.

[162] The Japanese Society of Gastroenterology. Evidence-based clinical practice guidelines for livercirrhosis. 2015.

[163] The Joint Committee on Guidelines for the Management of Stroke. Japanese Guidelines for the management of stroke 2015. 2015.

[164] Research Committee on Autoimmune Disease of the Ministry of Health, Labour and Welfare of Japan. Clinical Practice Guideline for the management of Polymyositis and Dermatomyositis. 2015.

[165] Japanese Society of Neurological Therapeutics, Japanese Society of Neurology, Japanese Society for Neuroinfectious Diseases. Clinical Practice Guideline for Bacterial meningitis. 2015.

[166] The Japanese Society for Tuberculosis. Clinical Practice Guideline for tuberculosis. 2015.

[167] Japanese Society of Psychosomatic Pediatrics. Clinical Practice Guideline for Psychosomatic Medicine in Children. 2015.

[168] The Japan Diabetes Society, The Japanese Society for Pediatric Endocrinology. Clinical Practice Guideline for Management of Diabetes in Children and Puberty. 2015.

[169] The Japanese Society of Child Neurology. Clinical Practice Guideline for Febrile Convulsion. 2015.

[170] Japanese Society for Inherited Metabolic Disease. Clinical Practice Guideline for Neonatal Mass Screening Target Diseases. 2015.

[171] Japanese Society of Cancer Nursing, Japanese Society of Medical Oncology, Japanese Society of Pharmaceutical Oncology. JSCN/JSMO/JASPO Joint Guidelines for Safe Handling of Cancer Chemotherapy Drugs. 2015.

[172] Japan Society of Clinical Oncology. Clinical Practice Guideline for proper use of antiemetic. 2015.

[173] Japanese Breast Cancer Society. Evidence-based clinical practice guidelines for breast cancer 1, Treatment. 2015.

[174] Japanese Breast Cancer Society. Evidence-based clinical practice guidelines for breast cancer 1, Epidemiology and Diagnosis. 2015.

[175] The Japanese Society of Pathology. Guideline for Pathological Diagnosis of Stomach Cancer and Breast Cancer HER2. 2015.

[176] The Japan NeuroEndocrine Tumor Society. Clinical Practice Guideline for Pancreatic and Gastrointestinal Neuroendocrine Tumor. 2015.

[177] The Japanese Urological Association. Clinical Practice Guideline for bladder cancer. 2015.

[178] The Japanese Urological Association. Clinical Practice Guideline for　Testicular Cancer. 2015.

[179] The Japan Society of Gynecologic Oncology. Clinical Practice Guideline for ovarian cancer. 2015.

[180] The Japan Society of Gynecologic Oncology. Clinical Practice Guideline for Vulvar Cancer and Vaginal Cancer. 2015.

[181] Japanese Society of Medical Oncology. Clinical Practice Guideline for Bone Metastasis. 2015.

[182] The Japanese Dermatological Association, Japanese Skin Cancer Society. Evidence-based Clinical Practice Guidelines for Skin Cancer. 2015.

[183] Japanese Hernia Society. Clinical Practice Guideline for Inguinal Hernia. 2015.

[184] The Japanese Orthopaedic Association. Clinical Practice Guideline for Cervical Spondylotic Myelopathy. 2015.

[185] The Japanese Orthopaedic Association. Clinical Practice Guideline for Prevention of Surgical Site Infection (bone and joint). 2015.

[186] Japan Osteoporosis Society, The Japanese Society for Bone and Mineral Research, Japan Osteoporosis Foundation. Clinical Practice Guideline for prevention and management of osteoporosis. 2015.

[187] Japan Society of Plastic and Reconstructive Surgeons, Japan Society for Surgical Wound Care, Japan Society of Cranio-Maxillo-Facial Surgery. Clinical Practice Guideline for Plastic Surgery on Skin Disease. 2015.

[188] Japan Society of Plastic and Reconstructive Surgeons, Japan Society for Surgical Wound Care, Japan Society of Cranio-Maxillo-Facial Surgery. Clinical Practice Guideline for Plastic Surgery on Acute Wound and Scar Keloid. 2015.

[189] Japan Society of Plastic and Reconstructive Surgeons, Japan Society for Surgical Wound Care, Japan Society of Cranio-Maxillo-Facial Surgery. Clinical Practice Guideline for Plastic Surgery on Chronic Wound. 2015.

[190] Japan Society of Plastic and Reconstructive Surgeons, Japan Society for Surgical Wound Care, Japan Society of Cranio-Maxillo-Facial Surgery. Clinical Practice Guideline for Plastic Surgery on Congenital Cranio-Maxillo-Facial Disease. 2015.

[191] Japan Society of Plastic and Reconstructive Surgeons, Japan Society for Surgical Wound Care, Japan Society of Cranio-Maxillo-Facial Surgery. Clinical Practice Guideline for Plastic Surgery on Acquired Cranio-Maxillo-Facial Disease. 2015.

[192] Japan Society of Plastic and Reconstructive Surgeons, Japan Society for Surgical Wound Care, Japan Society of Cranio-Maxillo-Facial Surgery. Clinical Practice Guideline for Plastic Surgery on Head and Neck and Facial Disease. 2015.

[193] Japan Society of Plastic and Reconstructive Surgeons, Japan Society for Surgical Wound Care, Japan Society of Cranio-Maxillo-Facial Surgery. Clinical Practice Guideline for Plastic Surgery on Trunk and Limb Disease. 2015.

[194] Japanese Society of Allergology. Guidelines for the management of atopic dermatitis. 2015.

[195] Research Committee on Progressive Kidney Injury of the Ministry of Health, Labour and Welfare of Japan. Evidence-based Clinical Practice Guideline for Rapidly Progressive Glomerulonephritis (RPGN). 2015.

[196] Research Committee on Progressive Kidney Injury of the Ministry of Health, Labour and Welfare of Japan. Evidence-based Clinical Practice Guideline for Nephrotic Syndrome. 2015.

[197] Research Committee on Progressive Kidney Injury of the Ministry of Health, Labour and Welfare of Japan. Evidence-based Clinical Practice Guideline for IgA Nephropathy. 2015.

[198] Research Group on Establishment of Clinical Practice System to Realize Reduction of Introduction of New Dialysis by Optimization of Chronic Renal Failure Diagnosis. Clincal Practice Guideline for CKD stage G3b to 5. 2015.

[199] The Japanese Continence Society. Clinical Practice Guideline for Overactive Bladder. 2015.

[200] Japan Society for the Study of Hypertension in Pregnancy. Best practice guide 2015 for care and treatment hypertension in pregnancy. 2015.

[201] Japan Otological Society, Japan Society for Pediatric ORL. Clinical Practice Guideline for Exudative Otitis Media in Children. 2015.

[202] Clinical Practice Guideline Development Group for Management of Allergic Rhinitis. Practical guideline for the management of allergic rhinitis in Japan <PG-MARJ>. 2015.

[203] Japanese Society of Periodontology. Clinical Practice Guideline for　Periodontal Treatment of Diabetic Mellitus Patient. 2015.

[204] Japanese Society of Dentistry for Medically Compromised Patient, Japanese Society of Oral and Maxillofacial Surgeons, Japanese Society of Gerodontology. Evidence-based Clinical Practice Guideline for Tooth Extraction of Antithrombotic Therapy Patient. 2015.

[205] Japanese Society of Oral Care. Clinical Practice Guideline for Oral Care in Hematopoietic Cell Transplant Patient. 2015.

[206] National Public University Hospital Council for Infection Control. Clinical Practice Guideline for Management of Hospital Infection. 2015.
